# To restrict or not to restrict? Practical considerations for optimizing dietary protein interactions on levodopa absorption in Parkinson’s disease

**DOI:** 10.1038/s41531-023-00541-w

**Published:** 2023-06-24

**Authors:** C. Rusch, R. Flanagan, H. Suh, I. Subramanian

**Affiliations:** 1 Food Science and Human Nutrition Department, Center for Nutritional Sciences, University of Florida, Gainesville, FL USA; 2 Department of Neurology, Norman Fixel Institute for Neurological Diseases, University of Florida, Gainesville, FL USA; 3 My Moves Matter, Dublin, Ireland; 4 Parkinson’s Disease Research, Education, and Clinical Center, Greater Los Angeles Veterans Affairs Medical Center, Los Angeles, CA USA; 5 Department of Neurology, David Geffen School of Medicine, University of California Los Angeles, Los Angeles, CA USA

**Keywords:** Parkinson's disease, Drug delivery

## Abstract

Administration of levodopa for Parkinson’s disease (PD) has remained the most effective therapy for symptom management despite being in use for over 50 years. Advancing disease and age, changing tolerability and gastrointestinal (GI) dysfunction may result in change in dietary habits and body weight, as well as unpredictable motor fluctuations and dyskinesias. Dietary proteins which convert into amino acids after digestion are implicated as major factors that inhibit levodopa absorption. For people living with PD (PwP) who experience motor fluctuations, low protein diets (LPD) and protein redistribution diets (PRD) may be effective and are often recommended as a non-pharmacologic approach for improving levodopa bioavailability. However, there is a lack of consensus on a standard definition of these diets and appropriate treatment algorithms for usage. This may be due to the paucity of high-level evidence of LPD and PRD in PwP and whether all or specific subgroups of patients would benefit from these strategies. Managing diet and protein intake with proper education and monitoring may reduce complications associated with these diets such as dyskinesias and unintentional weight loss. Additionally, alterations to medications and GI function may alter levodopa pharmacokinetics. In this narrative review we focus on 1) mechanisms of dietary protein and levodopa absorption in the intestine and blood brain barrier, 2) dietetic approaches to manage protein and levodopa interactions and 3) practical issues for treating PwP as well as future directions to be considered.

## Introduction

Often utilized early and throughout the course of Parkinson’s disease (PD), orally administered levodopa (3,4-dihydroxy-L-phenylalanine) is a precursor for dopamine and considered to be the standard pharmacologic treatment for management of PD symptoms^1,2^. While levodopa has been investigated in human studies as early as the 1960s^3,4^, optimization of delivery and absorption remain critical issues – particularly in people living with PD (PwP) who experience suboptimal improvements in motor symptoms and advanced disease. Levodopa (unlike peripheral dopamine) is transported via the gastrointestinal (GI) tract, enters systemic circulation and is eventually transported across the blood-brain-barrier (BBB) where it is later converted to dopamine for utilization. To improve absorption and prevent early (peripheral) conversion to dopamine, levodopa is usually co-administered with carbidopa or benserazide^5^. Furthermore, levodopa is catabolized by the enzyme catecholamine-*O*-methyltransferase (COMT) to form 3-*O*-methyldopa (3-OMD) which may inhibit levodopa metabolism to form dopamine. An increase in plasma levodopa levels has been documented with matched motor response in PwP^6,7^ and remains a useful tool for studying levodopa pharmacokinetics. Other factors that have been associated with levodopa bioavailability include age, sex, race, and low body mass index (BMI), which highlights the importance of dose customization^8–11^.

Adverse effects of chronic levodopa usage and disease progression include dyskinesias and increased motor fluctuations which can be defined as unpredictable changes from optimal motor control (“ON” period) to decreased motor control in which symptoms reappear (“OFF” periods)^12^. Levodopa-induced dyskinesias may appear as early as within the first 6 years from PD diagnosis^13,14^. These dyskinesias and motor fluctuations can also be accompanied by a host of issues including hypermetabolism, fatigue, dysphagia, low appetite and BMI, and decreased quality of life^14,15^. Disease progression may also contribute to the need for increased amounts and frequency of levodopa doses due to an elevated “threshold” for therapeutic response after chronic usage and a decrease in the amount of functional dopaminergic neurons^16^. Chronic levodopa absorption is hindered by complex interactions between drug kinetics (including patient-specific parameters), PD pathophysiology (disease progression and GI dysfunction) and food intake (e.g., dietary protein) which can prove challenging for management^5^. Dietary approaches such as low protein (LPD) and protein redistribution diets (PRD) have been proposed over the years to improve levodopa response. Efficacy of LPD and PRD diets have been systematically reviewed within the last decade^17,18^ but questions remain regarding appropriate treatment algorithms and customization of care to identify those PwP who would benefit the most from these diets. The purpose of this narrative review is to discuss 1) current literature on levodopa and dietary protein pharmacokinetics and interactions, 2) efficacy of LPD and PRD and 3) challenges and limitations for managing these interactions.

### Intestinal levodopa and dietary protein absorption

In the peripheral tissues (e.g., GI tract and systemic circulation), there is increased metabolism of levodopa to dopamine by the vitamin B6-dependent enzyme, aromatic L-amino acid decarboxylase (AADC). To prevent early conversion to dopamine in the peripheral tissues by AADC, AADC inhibitors (e.g., carbidopa or benserazide) are co-administered with levodopa to improve bioavailability, thus decreasing the cumulative therapeutic dose and subsequent degree of peripheral side effects^5^. When administered, levodopa is considered to be a large neutral amino acid and structurally similar to other aromatic amino acids such as phenylalanine, tyrosine, and tryptophan, and its absorption primarily occurs in the proximal intestine^19,20^.

The rate of gastric transit and sex differences can pose significant challenges to levodopa delivery. GI dysfunction is a common non-motor symptom in PD and highly prevalent in up to 80% of PwP. Constipation and gastroparesis (delayed gastric emptying) are among the more common GI symptoms in PwP^21^. While fasting levodopa time to peak absorption (*t*_max_) is rapid ranging from 15 to 60 min^22,23^, delayed gastric emptying and constipation can significantly increase the *t*_max_ and decrease the maximum peak concentration (*C*_max_) of the drug^5^. This reduction in *C*_max_ is possibly due to increased pre-systemic decarboxylation of levodopa by AADC, reducing bioavailability and absorption. While PwP are predominately male^24^, pharmacokinetic studies have shown that women have a higher area under the curve (AUC) and *C*_max_ even after adjusting for body weight^9,10,25^. It is posited from these studies that differences may be due to variations in hormones (i.e., estrogen status), body weight/BMI and composition, and COMT activity. Subsequently, dyskinesias are more prevalent in women likely due to higher levodopa bioavailability^26–28^.

Unlike levodopa, dietary forms of protein must undergo enzymatic digestion in the stomach and proximal intestine to unfold quaternary, tertiary and secondary structures prior to absorption in the proximal intestine^29^. These series of reactions yield mainly di- and tripeptides that can transport via intestinal peptide transporter 1 (PEPT1) on the apical membrane where they are further broken down in the enterocyte into free amino acids by cytosolic peptidases^30^. Free amino acids circulating in the lumen can enter the enterocyte at the apical membrane and exit on the basolateral side through these amino acid transport systems^29,31^. Amino acid transport systems are highly specific depending on affinity, and competition can arise during absorption. These transport systems are the primary target for levodopa absorption across the intestine and through the BBB.

**Fig. 1:**
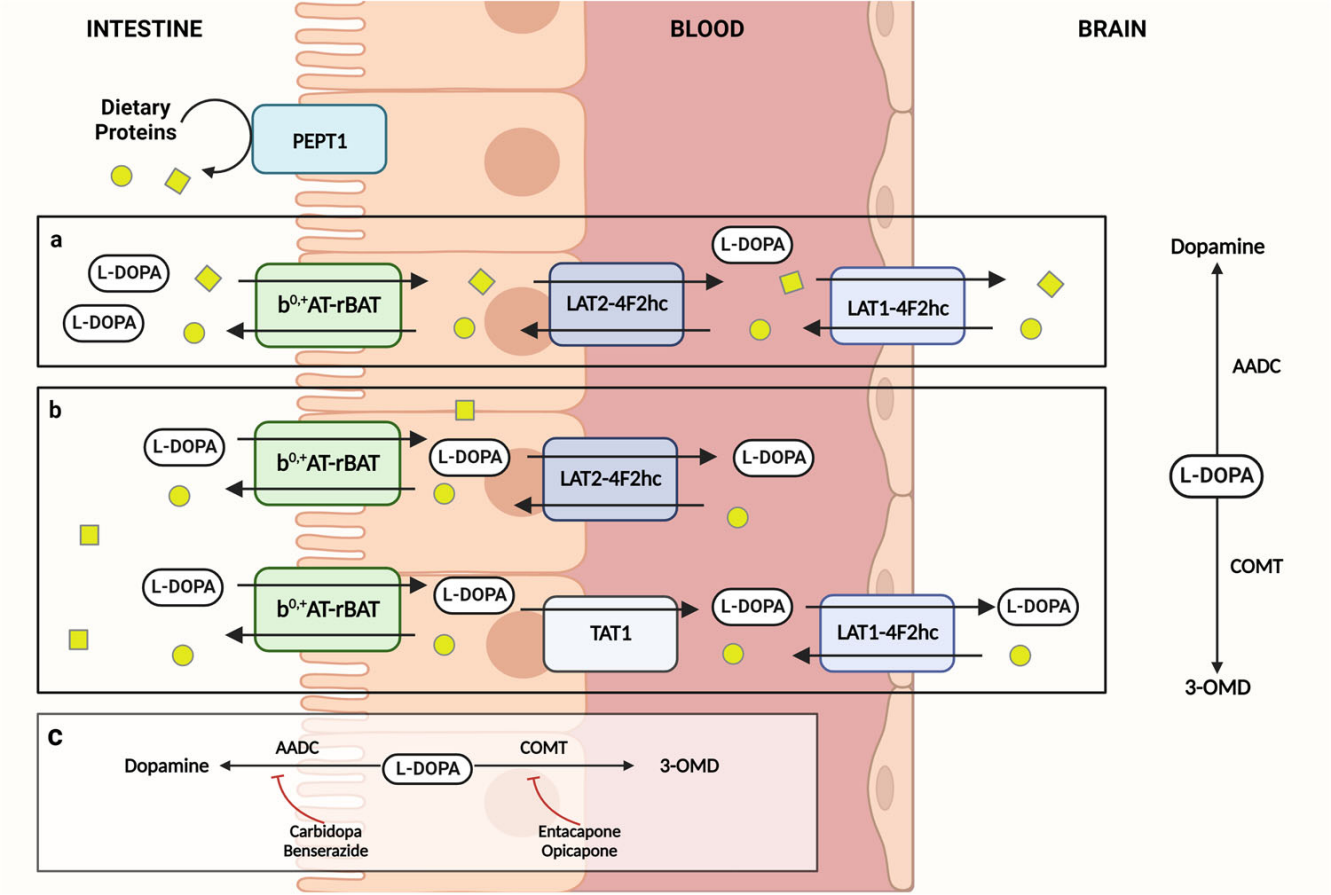
Transport of levodopa and free amino acids across the intestine, blood, and brain. Dietary proteins are broken down to free amino acids (yellow circles and squares) may compete with levodopa absorption. **a** When taken with meals, levodopa is competitively inhibited by neutral and cationic amino acids for uptake by b^0,+^AT-rBAT (SLC7A9-SLC3A1) decreasing transport across enterocytes by LAT2-4F2hc (SLC7A8-SLC3A2). After entering systemic circulation, transport across endothelial cells of the BBB by LAT1-4F2hc (SLC7A5-SLC3A2) may be reduced due to amino acid competition. **b** When taken on an empty stomach, levodopa is transported by b^0^,+AT-rBAT (SLC7A9-SLC3A1) and exits the basolateral side primarily by TAT1 (SLC16A10) and LAT2-4F2hc (SLC7A8-SLC3A2). Levodopa enters systemic circulation and transported across the BBB by LAT1-4F2hc (SLC7A5-SLC3A2) where it is metabolized into dopamine or 3-OMD. **c** Early peripheral metabolism of levodopa is prevented by co-administration of AADC and/or COMT inhibitors. L-DOPA, levodopa; AADC, aromatic L-amino acid decarboxylase; COMT, catecholamine-*O*-methyltransferase; 3-OMD, 3-*O*-methyldopa; SLC, solute carrier family; BBB, blood brain barrier.

In practice it has been thought that levodopa is absorbed by the same transporters as free, large neutral amino acids; however, current evidence remains limited on the intestinal transporters involved in this mechanism. To date, only one study published in 2014 has identified the transporters involved^32^. Using in vitro and in vivo models, Camrago et al. demonstrated levodopa can be transported by enterocytes across the apical membrane through the b^0,+^AT-rBAT (SLC7A9-SLC3A1) and exits the basolateral side primarily by TAT1 (SLC16A10) and LAT2-4F2hc (SLC7A8-SLC3A2) to a lesser extent^32^. b^0,+^AT-rBAT is an antiporter exchanging large neutral amino acids on the luminal side, while TAT1 is an aromatic amino acid uniporter and LAT2-4F2hc is antiporter on the basolateral side. In these models, levodopa is competitively inhibited by neutral and cationic amino acids such as leucine and arginine for uptake by b^0,+^AT-rBAT (Fig. 1). Earlier reports have suggested levodopa is only absorbed at the duodenum and proximal jejunum^33^. However, Camrago et al. demonstrated levodopa may be transported equally across the entire small intestine in in vitro models. Interestingly, a recent in silico computational model for levodopa kinetics proposed amino acid affinities of these transporters with levodopa having among the lowest affinity^34^. Future studies should look to confirm these findings in vivo to better understand the absorption and affinity for levodopa in comparison to other large neutral and cationic amino acids. The presence of AADC inhibitors (i.e. carbidopa and benserazide) or COMT inhibitors (i.e. entacapone) does not seem to inhibit levodopa transport across b^0,+^AT-rBAT despite having a similar structure to levodopa. In the postprandial phase, increased amino acid accumulation in the portal vein may trans-stimulate increased efflux from LAT2-4F2hc on the basolateral membrane, suggesting a potential target for improving levodopa absorption with meals.

The effects of meals and dietary protein on levodopa absorption has been described as early as 1973 in PwP who experience motor fluctuations^22,23,35–38^. The results of these studies further prompted investigations of protein-restricted diets in PwP (described later) in order to possibly limit competition of amino acid transport systems. Protein intake in both people with- and without PD has failed to show reduced *t*_max_ and *C*_max_ in previous studies^39,40^, which suggests that intestinal absorption of levodopa may be more affected by GI dysfunction (discussed in later sections) and fluctuations in plasma LNAA levels for transport across the BBB.

### Levodopa and amino acid competition at the blood-brain barrier

The bioavailability of levodopa within systemic circulation prior to crossing the BBB is considered one of the main determinants of levodopa pharmacokinetics^5^. Circulating amino acids (both endogenous and exogenous) can be transported across the BBB by a variety of amino acid transport systems^41^. Similar to intestinal absorption, levodopa has been shown to cross the BBB via the sodium-dependent antiporter, LAT1-4F2hc (SLC7A5-SLC3A2) expressed on endothelial cells^42,43^ (Fig. 1). Once levodopa crosses the BBB via LAT1, AADC functions to convert levodopa to dopamine, ultimately activating the dopaminergic systems within the brain. The transport rate of LAT1 has been shown to be influenced by the presence of intracellular amino acids as well as presence of thyroid hormone and other drugs such as gabapentin^44^. LAT1 does differ from other LAT transporters in the peripheral tissues (i.e., LAT2) as it displays even greater affinity for amino acids such as phenylalanine, tryptophan and leucine. These amino acids are found commonly in protein-containing foods and supplements which may drive further competition for levodopa absorption when administered with meals^45^.

The effect of plasma amino acid concentration (with or without meals) and inhibition of levodopa at the BBB has been described in 4 studies using both animal models and clinical studies in PwP^39,40,46–49^. Using MPTP-induced parkinsonian monkeys, Alexander et al. investigated the effect of levodopa and LNAA influx comparing striatal extracellular fluid (ECF) as a ratio to plasma levodopa^46^. As expected, co-infusion of LNAA and an oral high protein meal (given prior to infusion) inhibited uptake of levodopa by 54–75% and 17–56%, respectively. Differences were not seen between control and MPTP-induced monkeys suggesting the transport rate may not always be disease-specific. Two studies have measured levodopa and amino acid competition using positron emission tomography (PET) and demonstrated co-administration of amino acids led to a reduction of levodopa uptake compared to fasting in humans and plasma LNAA concentrations were negatively correlated (*r*^2^ = 0.51) with BBB influx rates in monkeys^47,48^. It should be noted that timing of dietary protein interventions in all studies was either via co-infusion or before levodopa administration. Because half-life of levodopa in both plasma and striatal ECF were noted to be approximately 30 min, future studies should aim to investigate carry over effects of dietary protein interactions when levodopa is administrated in a fasted state.

### Dietetic approaches for levodopa and protein interactions

Levodopa is often recommended to be taken on an empty stomach 20–30 min before or 1–2 h after meals to improve bioavailability^5^ (Box 1). The rationale for this timing is due to the increased likelihood for delayed transit time and competition with large neutral amino acids when taken with meals, as this may result in a significant decrease in peak plasma concentrations of 30% on average^22^. Because similar mechanisms for levodopa transport also occur at the BBB, this competition may also contribute to post-prandial motor fluctuations^40,50^. In PD, large cross-sectional studies have reported average protein intake is around 1.2 g/kg/day (1.5-fold higher than the Recommended Dietary Allowances [RDA])^51^ and correlated with a higher daily levodopa dose^52,53^. For PwP who experience significant motor fluctuations, the American Academy of Neurology (AAN) and European Society for Clinical Nutrition and Metabolism (ESPEN) have included LPD and PRD as potential complementary therapeutic diets for PD management in their guidelines^54–56^.

#### Low protein diets (LPD)

There is no standard definition adopted for LPD but is generally accepted as limiting protein consumption below the RDA for adults ( <0.8 g/kg of body weight per day)^51^. With the exception of 2 studies^37,57^, the use of LPD has not been thoroughly investigated. Both trials were single arm, controlled feeding studies consisting of multi-phase interventions including low protein (10 g/day or 0.5 g/kg body weight), normal protein (1.0 g/kg body weight) or high protein (2.0 g/kg body weight). In the study by Gillespie et al. the multi-phase interventions were implemented with either levodopa alone or levodopa combined with an unspecified peripheral inhibitor^37^. Timing of levodopa with meals was not reported. The authors noted body weight did not significantly differ during the interventions (data unreported). Participants (*n* = 8) reported discomfort consuming 10 g/day and 2.0 g/kg body weight while other diet phases including 0.5 g/kg/day were acceptable. Authors concluded the LPD tended to “potentiate and stabilize the therapeutic effect of levodopa” but no motor disability scales or scores were reported. In general, prolonged protein intake of 10 g/day is not recommended due to inability to satisfy whole body protein turnover and increase risk of deficiency^51^.

Mena et al. whom further reported on protein intakes of 10 g/day and 0.5 g/kg body weight in 7 participants^57^. When compared to the run-in phase (1.0 g/kg body weight), both protein intakes of 10 g/day and 0.5 g/kg/body weight resulted in reduction of New York University Scale scores of approximately 8 and 3 points, respectively. Of the participants followed for 2 months (*n* = 4) to 1 year (*n* = 3), consuming protein intakes of 0.5 g/kg/body weight resulted in 70% of participants reported satisfaction with diet and 30% reporting no improvement. Limitations of these studies also include small sample sizes, missing data, lack of randomization and variability in response amongst participants. Conflicting consensus exists between the AAN and ESPEN guidelines with ESPEN not supporting use of LPD in the absence of high-quality trials^54–56^.

#### Protein redistribution diets (PRD)

PRD diets have been more extensively investigated in the literature than LPD^17,18^. Similar to LPD, there is no consensus for recommended amounts of protein in PRD, but redistribution is defined as limiting protein intake at breakfast and lunch with no quantitative restrictions of protein content at dinner. Early work by Pincus et al. described one of the first PRD protocols which aimed to characterize the effect of a low protein phase (7 g) during the daytime (8:00 am–4:00 pm) on levodopa pharmacokinetics when compared to a high protein phase (160 g) in 15 participants^58^. Food intake in the evening was not specifically reported. During the daytime, plasma LNAA were significantly lower in the low protein phase as well as increased sensitivity to levodopa and improvements in disability scores were observed.

Further, PRD diets on levodopa response in PwP have been documented in 10 studies^35,38,59–66^. Seven studies^38,61–66^ implemented interventions with total protein intake between 30 and 80 g/day. Only 3 studies^35,59,60^ implemented interventions with protein intake based on body weight between 0.8 and 1.0 g/kg/day. Redistribution in all studies was typically defined as protein intake between 0 and 10 g during the day (including breakfast and lunch) and the remaining amount of protein consumed at dinner. Control diets were absent in 6 studies^60–65^ and outcomes were compared to participants baseline values. The remaining studies^35,38,59,66^ with control diet phases included either normal and/or high protein diets with even distribution between meals. Duration of interventions was typically short ( <1 month) with one study investigating feasibility of PRD after 2 years^62^.

Lower and/or improvements in motor symptoms (as measured by clinical scales or amount of “ON/OFF” time) in PwP following a PRD ranged from 32 to 79% depending on the scale used^35,38,60–62,64^. Barichella et al. reported significant differences between PRD and balanced protein diets on postprandial ON and OFF periods (mean difference approximately 30 and 107 min, respectively)^59^. Lowers disability scores with PRD were also reported in 3 studies^60,62,66^. Plasma concentrations of levodopa and LNAA after a PRD were also reported^35,38,65,66^. Higher concentrations of LNAA were significant in the high protein vs PRD groups^35,66^. However, there is conflicting evidence whether PRD improves plasma levodopa concentrations as studies reported either no difference between groups^35,38^ or higher concentrations with high protein diets^66^. In a separated study by Simon et al. levodopa pharmacokinetics were compared between a low protein breakfast (mean intake of 7.6 g) with a high protein lunch (mean intake of 38.7 g)^40^. No differences in *t*_max_ or *C*_max_ were observed between groups and significantly higher AUC for the high protein lunch was noted. Post hoc analyses by the authors revealed this effect may be mediated by levodopa dosage and carry over effects of the low protein breakfast as the high protein lunch was correlated with higher trough concentrations. Therefore, it is hypothesized the benefit of PRD may be a result of improved competition at the BBB vs peripherally and should be confirmed in future studies.

Further, a limited number of studies have attempted to identify characteristics of PwP who may benefit the most from modifications in protein intake (diet responders). Usual protein intake prior to implementation of LPD or PRD may influence responsiveness to the intervention (higher vs. lower baseline protein intake) and should be considered. Some studies have reported the most robust benefits of modifying protein intake in PwP who have experienced either shorter duration of motor fluctuations or treatment^60,61,64^. Motor fluctuations are associated with younger age of onset, sex and advanced stages of disease (Hoehn & Yahr [H&Y] stage ≥3)^67–69^. Interestingly, an in silico GI computational model of levodopa pharmacokinetics demonstrated H&Y stage 3 and 4 may benefit the most from modifications in protein intake (LPD or PRD)^34^. LPD did not show any benefit for the predicted AUC, while PRD was predicted to increase AUC compared to fasted and LPD.

Long term adherence of PRD assessed by Karstaedt et al. appears feasible with 30 out of 43 participants (70%) reporting compliance to diet recommendations after 1–2 years. Adverse effects of PRD were increased prevalence of dyskinesia (requiring reduction of levodopa dosage)^60,62,66^ and weight loss^59,60,62,64^ which may or may not be desired. Therefore, implementation of PRD should be closely monitored with careful consideration of levodopa dosage, body weight, BMI and total energy and protein intake to reduce unwanted side effects. While benefits were reported for PRD in all studies, data should be interpreted with caution as only 2 out of 10 studies were randomized controlled trials^38,59^. Generalizability to the larger population of PwP is unknown at this time due to heterogeneous study designs and small sample sizes (*n* < 20). Therefore, benefits of this diet should be confirmed in larger, randomized controlled studies.

#### Body weight monitoring and protein intake

Unintentional weight loss and loss of lean body mass should be a consideration for long-term utilization of LPD and PRD in PwP. Low body weight and weight loss have been associated with higher levodopa dose per kilogram of body weight (increasing risks for dyskinesias) and faster decline in motor function, respectively^70,71^. Discontinuation of PRD due to weight loss was described in 2 studies^60,62^. However, Barichella et al. reported a PRD is safe when estimated energy requirements are met (−1.8% within 6 months), suggesting monitoring nutritional status may minimize unintentional weight loss of PRD^59^. Malnutrition may be present in up to 24% of PwP^72^ and correlated with markers of PD severity^73^ and quality of life^74^. PwP are at risk of weight loss and malnutrition due increased symptom-associated hypermetabolism (i.e., tremors, dyskinesias) and decreased caloric intake (i.e., hyposmia, cognitive impairment, GI dysfunction, slowness)^15,72^.

Nitrogen balance studies can assess whole-body catabolism vs. anabolism status using the ratio of estimated nitrogen intake and 24 h urinary nitrogen loss, but this has not been adequately investigated in PwP. One small pilot study reported an average protein intake of 1.1 g/kg/day resulted in net nitrogen loss in PwP compared to healthy controls, indicating increased risk for muscle tissue breakdown^75^. This is consistent with previous guidelines that suggest older adults may require protein intake as high as 1.5 g/kg/day to achieve nitrogen balance and promote adequate muscle protein synthesis^76^. However, these guidelines may not always be appropriate for PwP when considering medication pharmacokinetics and expert consensus is needed to determine appropriate protein intake in this population. Factors (i.e., age, sex, race, levodopa responsiveness, body weight and risk of dyskinesias) should be taken into consideration when determining appropriateness of diet modification. PRD would be ideally initiated when motor fluctuations first occur, prior to increasing levodopa dosages to reduce dyskinesia risks.

Box 1 Considerations for managing diet, medications and gastrointestinal issues in PwP *Dietary Assessment*

- Take levodopa on an empty stomach 30 min before or 2 h after a meal.
- Assess possible food-drug interactions, by recording at least a 1-day dietary recall that includes amount/type of foods and drinks consumed, timing of meals, medications, and motor fluctuations.
- If motor fluctuations present, consider appropriateness of PRD with protein intake at least 0.8 g/kg/body weight. Monitor body weight and dyskinesias.
- Avoid use of low-protein diets ( < 0.8 g/kg/day) to prevent loss of lean body mass.
- Administer levodopa with a small carbohydrate snack (i.e., crackers, toast, applesauce, etc.) to reduce nausea after administration.
- High fiber diet and adequate hydration for constipation management to improve levodopa bioavailability.
- Low-fat diet for gastroparesis management to improve levodopa bioavailability.
- Consider dietitian referral for diet assessment and education for PwP.

*Medication Dosing and Timing*

- Educate PwP/care partners on indication, dose, frequency, administration times, what to do if missed dose, food and drug interactions, and potential adverse effects.
- Consider other formulations of levodopa delivery such as half tablets, extended-release orally disintegrating, transdermal or enterally administered to improve levodopa bioavailability.
- To prevent nausea, carbidopa may reduce side effects if available. A minimum daily dose of 75 mg is required to prevent peripheral conversion. Domperidone or trimethobenzamide can also be considered.
- Monitor and adjust levodopa dosage when implementing interventions that improve bioavailability to reduce dyskinesias.
- Consider pharmacist referral for medication therapy management and education.

*Gastrointestinal Function*

- Conditions such as gastroparesis, SIBO and/or constipation may impair levodopa absorption.
- Change in diet habits due to poor oral health (masticatory difficulties), anosmia, ageusia, and dysphagia may challenge dietary interventions and increase risk for weight loss.
- Stool softeners, bulk-producing and osmotic laxatives and suppositories should be used as needed for constipation management to improve levodopa bioavailabilty.
- Consider speech language pathologist and/or gastroenterology referral(s).

*PwP* people living with Parkinson’s, milligrams, *g* grams, *kg* kilograms, *SIBO* small intestine bacterial overgrowth.

### Additional considerations to improve levodopa response

**Fig. 2:**
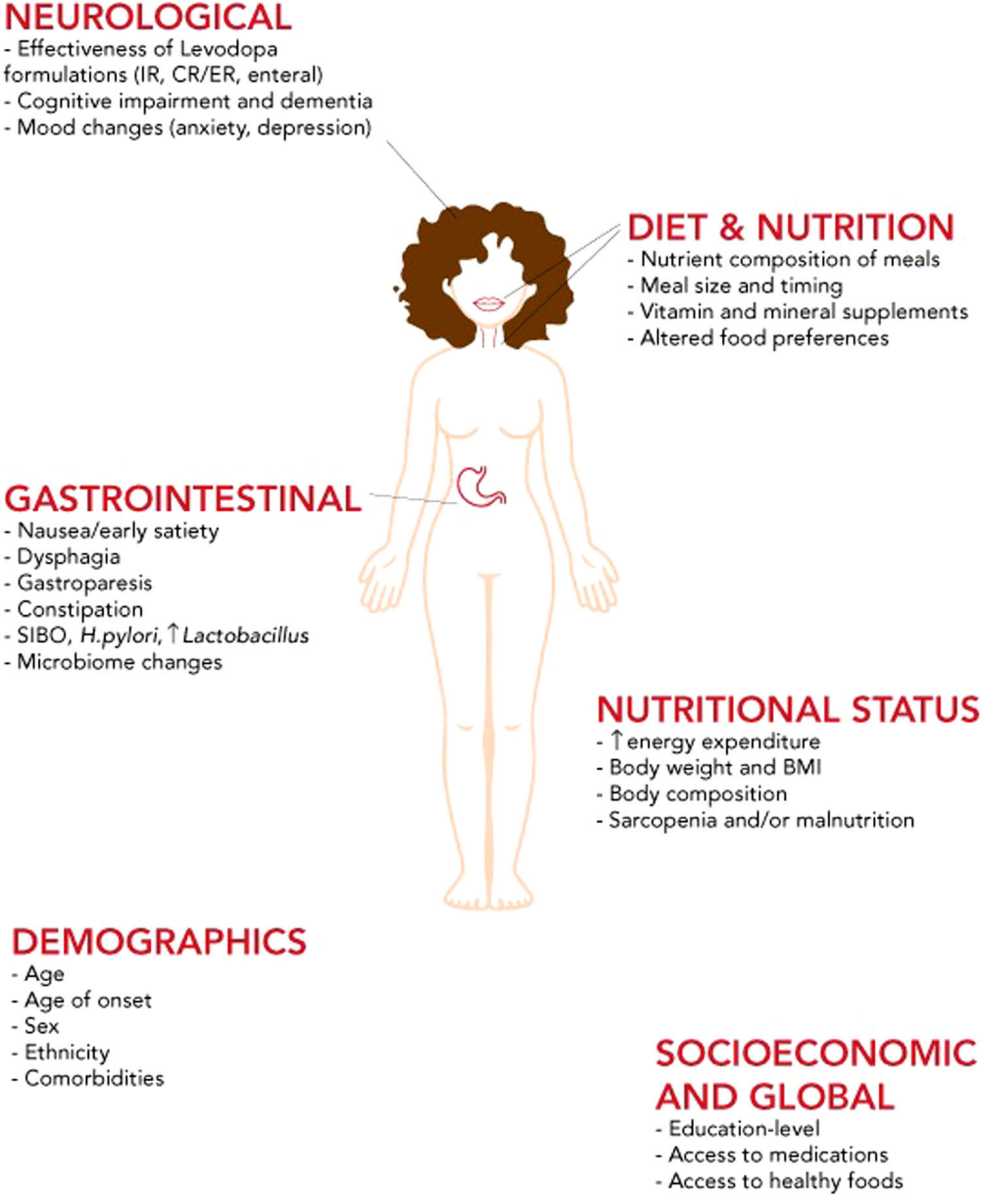
Factors that influence pharmacokinetics and response of levodopa. IR Immediate-release, CR Continuous-release, ER Extended-release, SIBO Small intestine bacterial overgrowth, *H.pylori Helicobacter pylori*, BMI Body mass index.

There are additional factors at play which affect the levodopa response of PwP that should be addressed in addition to dietary protein interactions (Box 1). These include understanding the importance of medication adherence and timing for PwP and their care partners. Similarly, an understanding of the impact of other nutrition-related and GI factors on symptom fluctuations should be addressed (Fig. 2). Consulting a gastroenterologist or various allied health professionals including pharmacists, dietitians, and speech/swallow pathologists may be considered.

#### Medication management and education

In addition to addressing dietary protein interactions with levodopa, optimization of medication therapy through effective medication education of PwP and their care partners, consistent medication adherence, and proper administration of medications may mitigate symptom fluctuations and improve quality of life in PwP. Helping with aids for compliance and working with a pharmacist with instructions tailored to cognitive and cultural context is key. When motor response to orally administered levodopa is suboptimal, alternative formulations (immediate vs. continuous vs. extended) and routes of administration (enteral vs. transdermal vs. inhaled) may be considered. These alternatives may compete differently with dietary proteins at both the intestine and BBB^36^. The amount of amino acid competition that occurs at the BBB with subcutaneous or inhaled levodopa has not been well established. The lack of competition with protein at the intestine and BBB may be a theoretical advantage for the choice of dopamine agonists in various formulations ranging from oral to patch to infusion (i.e., apomorphine pumps) over levodopa preparations and needs to be further investigated.

#### Gastrointestinal dysfunction

Beyond the current guidelines of taking levodopa on an empty stomach, PwP may still choose to take the drug with food to avoid GI side effects such as nausea and lightheadedness^77^. Some report difficulties with timing medication away from meals especially when levodopa dose frequency exceeds 3–4 times per day^78^. Exogenous factors that may influence gastric rate and levodopa bioavailability include pH-lowering medications (e.g., antacids and protein pump inhibitors)^79^ and gut microbial interactions such as *Helicobacter pylori* infections and small intestinal bacterial overgrowth^80^. Host-microbiome interactions are a growing area of research, including the responsiveness and delivery of levodopa medications in the GI tract. Certain species of gut bacteria (*Enterococcus faecalis* and *Lactobacillus brevis*) express tyrosine decarboxylase which can convert levodopa to dopamine peripherally and may contribute to heterogenous responses and side effects of levodopa administration^81,82^. Interestingly, investigations of genetically-engineered probiotic bacteria is an emerging area of research for improving levodopa delivery^83^. Nutrition interventions that aim to improve GI dysfunction such as constipation (high fiber diet and adequate fluids) and/or gastroparesis (low fat diet) may improve levodopa bioavailability^84^. Other GI symptoms such as anosmia, ageusia and dysphagia in PwP can complicate implementation of dietary interventions due to decreased appetite and reduced food and fluid intake^15^.

### Opportunities for future research

More investigations to support the customization of dietary protein in the management of PwP is of utmost importance. Levodopa absorption within the intestine facilitated by saturable transporters (e.g., b^0,+^AT-rBAT, TAT1 and LAT2-4F2hc) has been recently elucidated in vitro and in vivo. Future studies should confirm these findings with in vivo models and elucidate strategies for increasing trans-stimulation of levodopa by the transporter LAT2-4F2hc. While addressing dietary protein interactions with levodopa may aide in medication bioavailability, larger randomized, controlled clinical trials are warranted to clarify efficacy of PRD in subpopulations of different disease severity, ages, sex, ethnic, GI and genetic influences. Other dietary interventions such as plant-based diets (vegan or vegetarian) may also be a key area of investigation given plant-based proteins are lower in essential amino acids (i.e., leucine)^29^ that could compete with levodopa absorption. Future development of biomarkers or machine learning algorithms that can predict responsiveness of dietary protein modifications on levodopa pharmacokinetics could be helpful. Currently, dietary interventions for optimizing levodopa response in PwP are implemented only as a “rescue” therapy instead of utilizing a more holistic, proactive, and preventative therapeutic approach. Increased collaboration with allied health professionals and creation of dietary guidelines to manage diet in PwP among national organizations should be prioritized.

The efficacy and response to chronic levodopa treatment, coupled with dietary protein interactions remain a challenge for management of PD symptoms. PRD (and not LPD) may be an effective treatment approach for managing dietary protein interactions. Daily protein intake of 0.8 g/kg/body weight is adequate and routine assessment of motor symptoms and nutritional status could reduce complications of PRD. However, limitations exist within available evidence including absence of control groups, short trial durations and heterogenous study designs. Given these limitations, high-quality randomized, controlled trials are still warranted for PRD. Factors that should be considered in future research include disease stage and progression, dietary habits, body weight, duration of motor fluctuations, as well as the interactions of sex, race and genetic influences, co-medications & comorbidities. Medication management (including education) and GI dysfunction should also be addressed to improve levodopa pharmacokinetics, if warranted. This paper highlights the need to investigate and better understand the many underlying nutritional and pharmacokinetic factors before increasing levodopa dosing for PwP who experience motor fluctuations. Based on the current review, there are many gaps in knowledge that warrant timely attention until conclusive recommendations on dietary protein and PRD can be made for PwP.

### Reporting summary

Further information on research design is available in the Nature Portfolio Reporting Summary linked to this article.

## Supplementary information

NR Reporting Summary Checklist

## Data Availability

No datasets were generated or analyzed in this article.
